# Assessment of phylogenetic relationships and genetic diversity of *Sagittaria trifolia* using phenotypic traits and SNP markers

**DOI:** 10.1371/journal.pone.0302313

**Published:** 2024-06-03

**Authors:** Qun Ji, Feng Li, Xinfang Huang, Shuangmei Li, Zhixin Wang, Zhengwei Liu, Laichun Huang, Yingnan Yang, Honglian Zhu, Weidong Ke

**Affiliations:** Institute of Vegetables, Wuhan Academy of Agricultural Sciences, Wuhan, Hubei, China; USDA-ARS Southern Regional Research Center, UNITED STATES

## Abstract

The aquatic perennial herb *Sagittaria trifolia* L. commonly known as arrowhead, has been utilized in China both as a culinary vegetable and in traditional medicines. Characterizing the phylogenetic relationships and genetic diversity of arrowheads is crucial for improved management, conservation, and efficient utilization of the germplasm resources associated with this species. Herein, we presented the phenotypic traits and genome-wide DNA marker-based analyses of 111 arrowhead accessions, most of which were from China. Cluster analysis revealed that arrowhead could be categorized into two clusters based on 11 phenotypic traits, with Cluster 1 comprising two subclusters. All accessions were clustered into three sub-clusters based primarily on leaf shape and tuber weight. A set of 759,237 high-quality single-nucleotide polymorphisms was identified and used to assess the phylogenetic relationships. Population structure and phylogenetic tree analyses suggested that the accessions could be classified into two major groups, Group I was further subdivided into two subgroups, aligning with the clusters identified through morphological classification. By employing *Sagittaria lichuanensis* as an outgroup, the rooted tree revealed that the evolutionary relationships within the three groups followed a progression from Group I-1 to Group I-2 and finally to Group II. The landraces were clustered into one group along with the remaining wild accessions. The level of genetic diversity for Group I (π = 0.26) was slightly lower than that which was estimated for Group II (π = 0.29). The lowest pairwise differentiation levels (Fst, 0.008) were obtained from the comparison between groups I-2 and II, indicating that the two groups were the most closely related. This study provides novel insights into germplasm classification, evolutionary relationships, genomics and arrowhead breeding.

## Introduction

The genus *Sagittaria* L. (Alismataceae) comprises approximately 30 species that are widely distributed throughout tropical and temperate regions [1]. *Sagittaria* is a perennial aquatic herb which is naturally confined to the shallow waters, freshwater wetlands, pools and rice paddies [2]. *Sagittaria sagittifolia* is a vegetable commonly consumed in the Chinese daily diet. The corm of this plant is notably abundant in starch, carbohydrates, crude fiber, vitamins, proteins, fat, minerals, and choline [3, 4]. However, it is worth noting that *S*. *sagittifolia* has been classified under the name *Sagittaria trifolia* in both versions of the “Flora of China” [1, 5]. Throughout this manuscript, *S*. *trifolia* is the classification used to describe the arrowheads. Besides being included as fresh vegetables, products derived from *Sagittaria* are exported even outside of the typical season [6]. In addition, *S*. *trifolia* plant stalks along with by-products of food processing, have been employed as novel biosorbents for the adsorption of heavy metal ions. This application is significant for the sustainable utilization of arrowhead plants and contributes to addressing environmental issues associated with the disposal of abandoned plant stalks [7]. Arrowheads are recognized as valuable traditional medicinal plants owing to their health benefits, including low risk of skin diseases, rheumatism, indigestion, and headache [8].

*Sagittaria* is a complex taxon owing to its diverse morphology and widespread dispersal. In recent years, great progress has been made in investigating the genetic variation and genetic relationships within the genus *Sagittaria*, including *Sagittaria isoetiformis*, *Sagittaria teres*, *Sagittaria graminea* var. *graminea*, *Sagittaria graminea* var. *chapmanii*, *Sagittaria platyphylla*, *Sagittaria Lancifolia*, and *Sagittaria latifolia*, all of which have been sampled from North America and are a few representative species of *Sagittaria* [9–11]. Another recent study had focused on the phylogeny and biogeography of *Sagittaria* from Europe, Africa, Asia, North America, and South America. In that study, 74 accessions delimited to 29 species were sampled for chloroplast DNA and nuclear DNA (ITS) analyses, and the taxonomic status and biogeography of *Sagittaria* spp. in Alismataceae were investigated extensively in the study [12]. Nine species, one subspecies, one variety, and one variant of *Sagittaria* native to China are recorded in the Chinese version of the “Flora of China” [1]. In the revised English translation of the “Flora of China,” only seven *Sagittaria* species (two endemic) are recorded, and all species have a diploid number of 22 [5]. To date, phylogenetic studies have identified six species and one variety from China, including *Sagittaria guyanensis*, *Sagittaria lichuanensis*, *Sagittaria natans*, *Sagittaria potamogetifolia*, *Sagittaria pygmaea*, *Sagittaria trifolia* var. *trifolia* and *Sagittaria trifolia* var. *Senensis* [13–17]. Given the endangered and endemic status of two species, *S*. *lichuanensis and S*. *potamogetifolia* in China, our sampling efforts were confined to only a few relatively small populations with a limited geographical region. For example, Tan et al. sampled only 54 individuals from the six extant populations of *S*. *potamogetifolia*, to study the chloroplast DNA variation and phylogeographic patterns of this species using chloroplast DNA *atp*B-*rbc*L intergenic spacer sequence variation as molecular markers [13]. Genetic markers, such as biochemical and molecular markers, can help elucidate the genetic background of accessions, which can support subsequent analyses. Allozyme variations have been used to describe the patterns of genetic relationships within and among populations of *S*. *graminea* and *S*. *latifolia* [10, 11]. Recently, only a limited number of molecular genetic resources have been made publicly available for *Sagittaria*. Specifically, there are merely a few dozen *Sagittaria* DNA sequences available in the public DNA databases, such as the National Center for Biotechnology Information (NCBI) GenBank database and the DNA Data Bank of Japan (DDBJ). This scarcity renders the development of the application of molecular markers impractical. Chloroplast DNA *atp*B-*rbc*L intergenic spacer sequences [13, 18, 19], random amplified polymorphic DNA [20], restriction fragment length polymorphism [21], inter-simple sequence repeat [14, 15], and simple sequence repeat [16, 22, 23] have been used as markers to examine the sequence variation in *Sagittaria* populations. However, the number of aforementioned markers is limited. SNPs, being the most abundant and stable type of genetic marker, have been extensively generated through whole-genome resequencing [24] and various genome complexity-reduction protocols, including genotyping-by-sequencing [25], restriction site-associated DNA sequencing [26], and specific-length amplified fragment sequencing (SLAF-seq) [27]. SLAF-seq has been widely applied in many plants for various aspects of genetic/genomic research, including phylogenetic analysis [28], genome-wide association studies [29], high-density genetic map construction [30], and QTL mapping [31]. SLAF-seq is a valuable tool with the advantages of low cost and high throughput, particularly when plants or animals lack a reference genome. This high-resolution method has been applied to many plants without a reference genome, including Kenaf [32], *Picea crassifolia* [33], and *Elymus sibiricus* [34].

Approximately 111 accessions of *S*. *trifolia* have been preserved in the Wuhan National Germplasm Repository for Aquatic Vegetables, whose mission is to collect, preserve, evaluate, and utilize of aquatic vegetable resources. In the present study, we developed an integrated workflow by combining morphological traits and SLAF-seq-generated SNPs to evaluate phenotypic and genome-wide variations in arrowhead accessions collected from an extensive geographical area, encompassing China and its neighboring countries, including Thailand and North Korea. The genetic diversity and relationships among these accessions were evaluated for the sustainable use of *S*. *trifolia* resources.

## Materials and methods

### Plant material and DNA extraction

The plant material included 40 *S*. *trifolia* subsp. *trifloia* and 71 *S*. *trifolia* subsp. *leucopetala* accessions. All 40 *S*. *trifolia* subsp. *trifloia* accessions were wild resources, and 71 *S*. *trifolia* subsp. *leucopetala* accessions were landraces. When collecting *S*. *trifolia* resources in the field, specimens found in cultivated areas were classified as landraces. Those collected in wild habitats, distinct from cultivated fields, and exhibiting no evidence of escape from cultivated arrowhead, were recognized as wild resources. Thirty-eight wild accessions were collected from the natural distribution areas of China; one was found in Thailand and the other was identified in North Korea. Seventy-one landraces were located in the main distribution areas of China. Of the 111 accessions, 109 were from China, including 21 from Yunnan, 18 from Hubei, 18 from Guangxi, 13 from Anhui, 11 from Jiangsu, four each from Hebei, Henan, Jiangxi, Zhejiang, and Sichuan, three from Guizhou, two each from Guangdong and Shanghai, and one from Shaaxi (S1 Table). *S*. *lichuanensis*, collected from Fujian, was employed to root the phylogenetic tree. All 112 accessions were obtained from the Wuhan National Germplasm Repository for Aquatic Vegetables.

Arrowheads are capable of both sexual and asexual reproduction; however, they propagate asexually in cultivation and ex-situ conservation [3, 4]. Tubers were chosen as the reproductive organs in the present study and new plants were grown from the tuber apical buds. Flowers were removed before blooming to avoid biological confounders caused by seed germination. Plants of a specific accession were genetically identical in terms of asexual reproduction. Bulked young leaves from each accession were collected in the spring season, immediately frozen in liquid nitrogen, and then stored at −80°C until use. DNA was extracted using the DP305 DNA Extraction Kit (Tiangen, Beijing, China). The quality of the DNA was detected by agarose gel electrophoresis, and the DNA concentration was quantified using a NanoDrop-2000 spectrophotometer (Thermo Fisher Scientific, Waltham, MA, USA). The final DNA concentration was adjusted to 50 ng μl^-1^.

### Phenotypic traits

A total of 111 accessions were individually planted and vegetatively propagated in pottery jars at the Wuhan National Germplasm Repository for Aquatic Vegetables. Pottery jars (inner diameter, 56 cm; height, 40 cm) were arranged outside in rows and columns with a space of 60 cm × 60 cm. Each pottery jar was filled with soil to a depth of 25 cm, and the remaining space was filled with water to a depth of 15 cm.

In April 2019, six healthy tubers from one accession were planted in a pottery jar, and in June, all seedlings were removed, and three well-growing tubers were selected for replanting in the same jar. New plants developed from the rhizomes during the non-flowering stage. To avoid biological confounders, the continuously blooming flowers were destroyed before the seeds matured. During the flowering stage, key morphological and agronomical characteristics, plant height, petiole diameter, middle lobe length and width, and lateral lobe length and width were measured and recorded for three representative plants per accession. In January 2020, after the tubers matured, the soil was poured out from each jar, and all tubers in each jar were collected and kept together in a mesh bag. Tuber characteristics, tuber longitudinal and transverse diameters, tuber apical bud length and diameter, and tuber weight were measured and recorded for three representative tubers per accession [35]. The above planting and investigation processes were repeated over the subsequent three years. Phenotypic traits data was obtained in triplicate as biological replicates for each accession in three years. The cultivation environment has a significant impact on plant growth, and due to limitations in the availability of 3-m^2^ concrete ponds (2 m × 1.5 m), we were unable to plant all accessions. Therefore, 16 of the 111 accessions were planted in 3-m^2^ concrete ponds, simultaneously with the experiments in the jars. Except for the fact that 10 healthy tubers were planted first and 6 seedlings were retained, the other experimental procedures and data acquisition processes were similar to those in the jars.

Data processing, including univariate analysis of variance (ANOVA), correlation analysis, and cluster analysis, was performed using SPSS v. 22 (IBM Corp., Armonk, NK, USA). Clustering was performed using the hierarchical cluster method, utilizing 11 continuous variables as the basis for clustering.

### Specific-length amplified fragment library construction and sequencing

The specific-length amplified fragment (SLAF) library was constructed using the Sun protocol with minor modifications [36]. A pre-designed experiment was conducted to determine the optimal combination of restriction enzymes and fragment sizes to obtain optimal SLAF yields and maximum SLAF-seq efficiency. Candidate restriction enzymes were identified only if they avoided repeated SLAFs and yielded abundant high-quality SLAF tags that were evenly distributed across the genome of *S*. *trifolia*. Purified genomic DNA was digested by the most appropriate restriction enzyme combination of *Hpy166II* and *Scal-HF*, and *Oryza sativa* L. subsp. *japonica* ‘Nipponbare’ DNA was used as a control to assess the enzyme digestion efficiency, to guarantee the accuracy and validity of the procedure. To ensure sequence depth uniformity of the fragments, DNA fragments of 364–414 bp in size were retained and used for subsequent high-throughput paired-end sequencing on an Illumina HiSeq 2500 system (Illumina, Inc., San Diego, CA, USA) according to the manufacturer’s protocol at Beijing Biomarker Technologies Corporation.

Although all the fragments were added into a single lane, the sequence reads of each sample were separated based on their barcodes. After the adaptor was trimmed, all the reads were processed for stricter quality control and evaluation. For quality control, the GC content and ratio of high-quality reads with a quality value above Q30 (0.1% sequencing error) were estimated. Reads with low-quality bases or adapter contamination were discarded. All paired-end reads with clear barcodes were clustered based on sequence similarities, and sequences with over 90% identity were placed into the same cluster and recognized as a single SLAF tag. The sequence similarity of the same SLAF tag between different samples was much higher than that between different SLAF tags. SLAF tags with the highest sequencing depth were used as reference tags, and high-quality trimmed paired-end reads were mapped onto the reference tags using Burrows-Wheeler Aligner (BWA) software [37]. Alleles at each SLAF locus were determined using a minor allele frequency (MAF) assessment. As *S*. *trifolia* is diploid, the SLAF locus should contain no more than four tags. Thus, if one SLAF locus contained more than four tags, it was considered to contain repeated SLAF tags and was filtered out. SLAF tags with 2 to 4 alleles were deemed polymorphic and used for subsequent SNP development. SNP polymorphisms were identified using GATK [38] and Samtools [39] software using default parameters, respectively. Only the SNPs identified by both software programs were considered to be high quality and selected for future use.

### Analysis of population structure and genetic relationship

SNPs associated with a missing rate > 20% across the germplasm set were excluded. Similarly, SNPs with a minor allele frequency (MAF) < 5% were removed. Qualified SNPs were used for subsequent analyses. A total of 759,237 SNPs from 111 accessions were used to investigate population structure and genetic relationships. Population structure was analyzed using ADMIXTURE software with the maximum-likelihood method. Five independent runs were performed for each populations (*K*), limited to the range of 1–10. Accessions were allocated to a corresponding memberships within groups according to their maximum membership probabilities [40]. A phylogenetic tree was constructed using the MEGA5 package [41] with the neighbor-joining method, followed by a principal component analysis (PCA) conducted in Cluster v. 3.0 [42]. The Stacks populations program [43] was applied to calculate nucleotide diversity (π) and pairwise differentiation levels (Fst). Neiʼs genetic diversity index and Neiʼs genetic distance were calculated in the R packages “snpReady” and “StAMPP,” respectively [44, 45].

## Results and discussion

### Phenotypic differences among *Sagittaria trifolia* accessions

Based on key morphological and agronomic characteristics, plant height, petiole diameter, middle lobe length and width, lateral lobe length and width, tuber longitudinal and transverse diameters, tuber apical bud length and diameter, and tuber weight, the 111 accessions were divided into two clusters in the clustering analysis. Cluster 1 comprised 39 accessions, whereas Cluster 2 comprised the remaining 72 accessions. Cluster 1 was further categorized into two subgroups. Cluster 1.1 encompassed nine accessions and Cluster 1.2 comprised 30 accessions (S2 Table).

For all traits except middle lobe width and lateral lobe width, the average values of Cluster 1.2 accessions lay in between Cluster 1.1 and Cluster 2, and the differences were significant (P<0.05). In terms of middle lobe width and lateral lobe width traits, the average values were significantly higher in Cluster 2 in comparison to those in Cluster 1.1 or Cluster 1.2 (P<0.05). However, there was no significant in these two traits between Cluster 1.1 and Cluster 1.2 (Table 1).

**Table 1:** Summary of statistical information of phenotypic traits within the *Sagittaria trifolia* population.

Trait	Cluster 1.1 (n = 9)	Cluster 1.2 (n = 30)	Cluster 2 (n = 72)
**Plant height Mean (cm)**	31.19^a^	55.39^b^	71.40^c^
**Petiole diameter Mean (cm)**	0.59^a^	0.78^b^	1.14^c^
**Middle lobe length Mean (cm)**	4.07^a^	7.55^b^	9.55^c^
**Middle lobe width Mean (cm)**	4.55^a^	4.43^a^	8.45^b^
**Lateral lobe length Mean (cm)**	5.90^a^	10.07^b^	12.17^c^
**Lateral lobe width Mean (cm)**	2.24^a^	2.54^a^	4.64^b^
**Tuber longitudinal diameter Mean (cm)**	1.72^a^	2.60^b^	3.96^c^
**Tuber transverse diameter Mean (cm)**	1.33^a^	2.25^b^	2.87^c^
**Tuber apical bud length Mean (cm)**	2.69^a^	3.81^b^	5.27^c^
**Tuber apical bud diameter Mean (cm)**	0.40^a^	0.57^b^	0.86^c^
**Tuber weight Mean (g)**	3.88^a^	7.05^b^	15.40^c^

Groups with means that do not share a common letter are significantly different (LSD test for homogeneous variance or Tamhane’s test for non-homogeneous variance; α = 5%).

**Fig 1:**
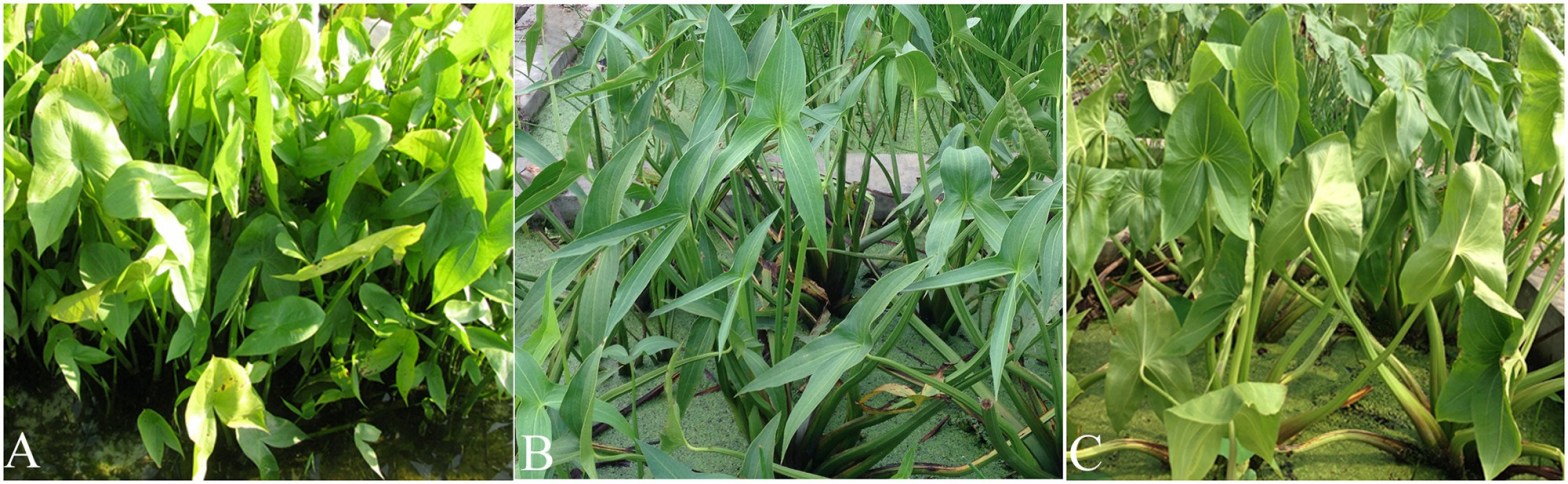
*Sagittaria trifolia* plants. From left to right: plants in clusters 1.1, 1.2, and 2.

**Fig 2:**
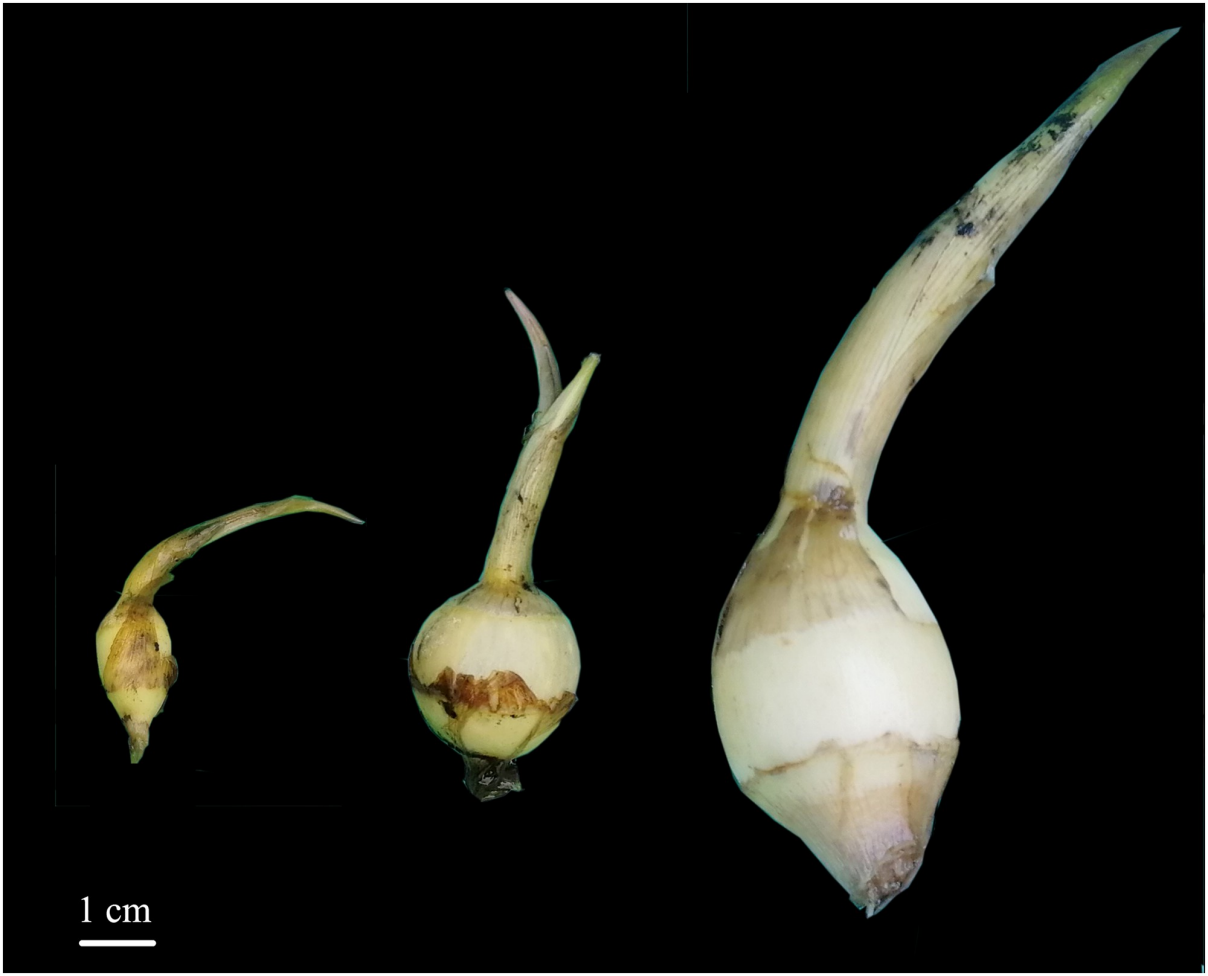
*Sagittaria trifolia* tuber phenotypes. From left to right: tubers of clusters 1.1, 1.2, and 2.

All the accessions (n = 9) in subgroup Cluster 1.1 could be distinguished from the other accessions (Fig 1). They had the following characteristics: short plant, compact plant shape, extremely strong branching ability, hastate leaves constricted between the middle and lateral lobes, green leaves lighter than those of other accessions, and small tubers (Fig 2). The mean for plant height, tuber longitudinal diameter, tuber transverse diameter,and tuber weight were 31.19 cm, 1.72 cm, 1.33 cm, and 3.88 g, respectively. All values were the lowest among the three groups (Table 1).

**Fig 3:**
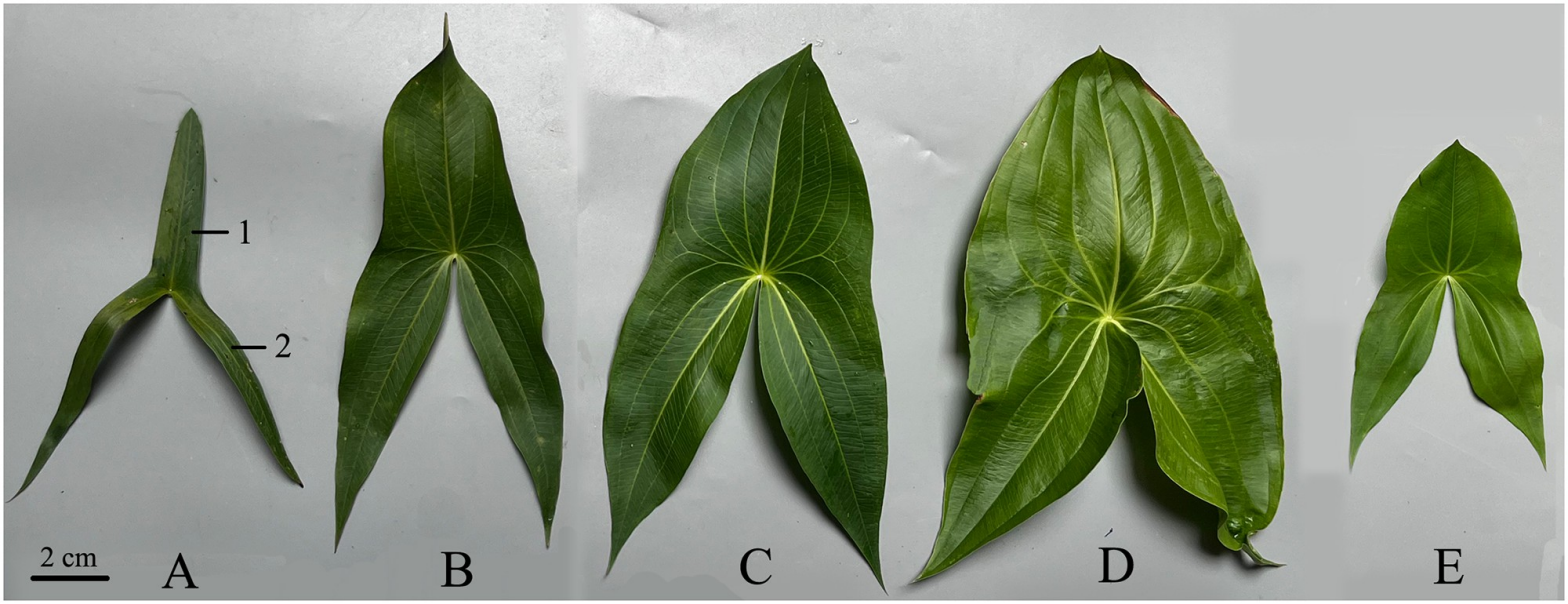
Different leaf shapes. (A) Narrowly sagittate leaf (0.17). (B) Sagittate leaf (0.30). (C) Sagittate leaf (0.39). (D) Broadly sagittate leaf (0.45). (E) Hastate leaf. The middle lobe and lateral lobes are indicated by numbers 1 and 2, respectively. Values in brackets are the lateral lobe width:length ratios of the arrow-shaped leaves.

The accessions of Cluster 1.1 had extremely strong branching ability and could produce a large number of rhizomes, the rhizomes arising from the axillary buds on underground nodes could develop into offshoots, resulting in dense plants with extremely small tubers (1.5–6.6 g planted in pottery jars in Wuhan) (Figs 2 and 3). However, the climate and cultivation conditions are very different in the Guangxi Province, and tubers can grow to larger sizes. The tubers exhibited a sweet and delicate flavor, distinct from the slightly bitter taste observed in the tubers of other accessions. Notably, these taste perceptions were subjective. Herein, four accessions of Cluster 1.1, collected from Guangxi Province, were landraces, the tubers of which were used for hot pots in Guangxi Province, and the hot pot was a dish of Chinese stew. The average tuber weight was greater in Cluster 1.2 in comparison to Cluster 1.1. Tubers were the main edible organ; therefore, several accessions (n = 5) with a high tuber weight were landraces. The average tuber weight in Cluster 2 was the largest among these clusters; therefore, most accessions in this cluster were landraces (n = 62) (Fig 2, S1 Table).

In our field investigation, we found that the diversity in leaf shape and tuber weight was very high. Therefore, leaf shape and tuber weight are the two most important traits. All accessions in Cluster 1.1 had hastate leaves constricted between the middle and lateral lobes, which were distinct from the arrow-shaped leaves of accessions in Cluster 1.2 or Cluster 2. However, the shapes of the arrow-shaped leaves differed significantly. Our phenotypic trait investigation revealed that leaf shape, indicated by middle lobe or lateral lobe width:length ratio, was more important than the separate middle lobe or lateral lobe width and length when classifying arrow-shaped accessions into clusters. The middle lobe width:length ratio was positively and significantly correlated with the lateral lobe width:length ratio, with a correlation coefficient of 0.773 (*P*<0.01). Compared with the middle lobe width:length ratio, the lateral lobe width:length ratio was consistent with the leaf shape determined by the subjective judgments of the researchers. Thus, the lateral lobe width:length ratio was used to distinguish the arrow-shaped leaves. The classification of arrow-shaped leaves was determined based on the lateral lobe width:length ratio. If the ratio was ≤ 0.27, the leaf was categorized as narrowly sagittate. For ratios falling within the range of 0.28 to 0.38, the leaf was classified as sagittate. If the ratio was ≥ 0.39, the leaf fell into the broadly sagittate category (Fig 3). Of the 30 aforementioned Cluster 1.2 accessions, 19 had narrowly sagittate leaves and 11 had sagittate leaves. The accessions in Cluster 2 possessed broadly sagittate leaves (n = 34) and sagittate leaves (n = 38) (S2 Table).

Some accessions in both clusters 1.2 and 2 had sagittate leaves; when an accession had sagittate leaves and a tuber weight of less than 12 g, it was converged to Cluster 1.2. Additionally, when an accession had sagittate leaves and a tuber weight of greater than 12 g, it converged to Cluster 2 (S2 Table). Therefore, although 11 phenotypic traits were used in the clustering analysis, it was found that all accessions of arrow-shaped leaves could be clustered into two categories using only two traits: lateral lobe width:length ratio and tuber weight.

In horticultural classification, *S*. *trifolia* were conventionally classified into cultivars and wild resources based on the size of their tubers, cultivars were mainly categorized as landraces. A previous attempt to classify the 67 *S*. *trifolia* accessions relied on 13 morphological traits. The samples included 41 landraces and 26 wild resources. All accessions were divided into three categories. The first category included 24 wild resources, the second category included 18 landraces and one wild resource, and the third category included 23 landraces and one wild resource. Accessions in Categories 2 and 3 were the main cultivars [46]. In the present study, 111 accessions were divided into two clusters; accessions in clusters 1 and 2 were corresponded to wild resources and landraces, respectively.

In the book “Descriptors and data standard for arrowhead *Sagittaria trifolia* L. var. *sinensis* (Sims) Makino”, the leaf shape of *S*. *trifolia* can be classified as narrowly sagittate, sagittate, or broadly sagittate, and the three standard leaf shapes were displayed in the book [35]. However, leaf shape is a continuous variable, and many accessions were intermediate between narrowly sagittate and sagittate, or between sagittate and broadly sagittate, making it difficult to determine the leaf shapes of these accessions. In the present study, the leaf shapes of the arrow-shaped accessions were determined by the lateral lobe width:length ratio, making the survey results more objective and unaffected by the researchers.

In the present study, all 111 accessions were planted in pottery jars; therefore, the values of 11 traits were lower than those obtained from the accessions cultivated in 3-m^2^ concrete ponds. Leaf shape and tuber weight are the most important parameters in the classification of *S*. *trifolia*. However, these traits were easily affected by the cultivation environment, and 16 accessions from the 111 accessions were selected for planting in 3-m^2^ concrete ponds, simultaneously with experiments in the jars. The range of lateral lobe width:length ratio and tuber weight were 0.28–0.53 and 9–22.5 g in pottery jars, however, when planted in the concrete ponds, the range improved to 0.36–0.74 and 46.9–85.9 g, respectively. As a result, if all the accessions were planted in the concrete ponds, the range of the lateral lobe width:length ratio and tuber weight would need redefining to distinguish all accessions.

### Characterization of specific-length amplified fragment sequencing data

Samples were collected from *S*. *trifolia* accessions originating from different regions of China and neighbouring countries. A total of 111 accessions were genotyped using SLAF-seq technology. About 297,776 expected high-quality SLAF tags evenly distributed across the genome of *S*. *trifolia* were predicted using a predesigned scheme. SLAF libraries of all 111 accessions were subjected to Illumina high-throughput sequencing. A total of 670 million 364–414 bp long paired-end reads were generated, with approximately 92.2% of the sequencing reads having a quality score above Q30. A mean GC content of 40.43% was obtained, which varied from 39.28 to 42.03% for the different accessions (S3 Table).

All reads were clustered into SLAFs based on sequence similarity. After quality filtering, 33,792,495 high-quality SLAFs containing 385.64 million reads, were identified. The number of reads for each accession ranged from 1,844,938 to 5,479,485, with a mean of 3,474,195. The number of SLAFs for each accession varied from 195,256 to 391,453, with a mean of 304,437. The sequencing depth of SLAFs for each accession ranged from 7.48 × to 14.78 ×, equivalent to a mean coverage of 11.37 × (S3 Table). In total, 1,743,977 of the high-quality SLAFs showed polymorphisms across 111 accessions. Out of the total number of 21,112,408 SNPs, 759,237 were selected as they met the thresholds: MAF≥0.05 and SNP integrity≥ 0.8.

Arrowheads propagate asexually in cultivation and ex-situ conservation. Heterozygous SNPs accounted for 6.04% of the polymorphic SNPs (S4 Table). It has a higher frequency of heterozygous SNP loci than water dropwort (3.58%), which reproduces asexually in practical application [47]. However, the heterozygous SNP ratio of arrowheads was significantly lower than that of apples (22.35%) [48]. In *S*. *trifolia*, asexual reproduction decreased heterozygosity to a greater extent than hybridization.

### Genetic relationship of the germplasm

**Fig 4:**
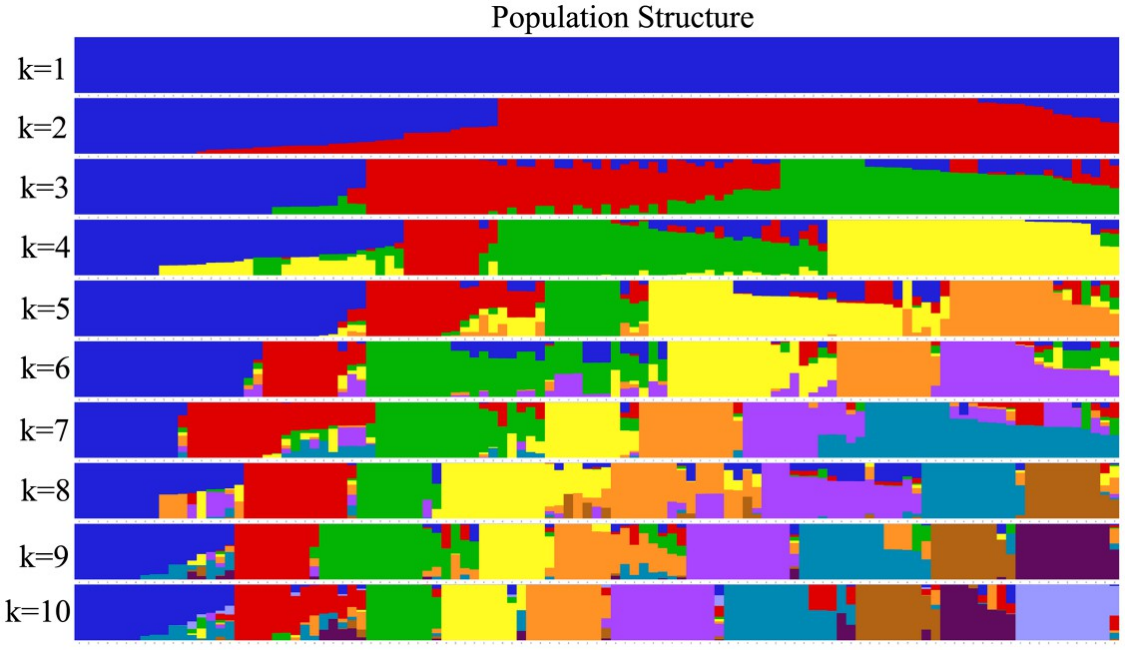
Population structure of 111 *Sagittaria trifolia* accessions generated using ADMIXTURE. Each color represents one population. The vertical bars represent individual accessions. The ratio of each colored component in each vertical bar represents the membership probability of accessions belonging to different populations.

The maximum likelihood of the estimated membership fractions of the 111 accessions was in the range of 1–10 for different K values, based on the set of 759,237 SNPs. An optimal K value of 2 was proposed, indicating that this population could be categorized into two groups: 45 and 66 accessions (Fig 4).

**Fig 5:**
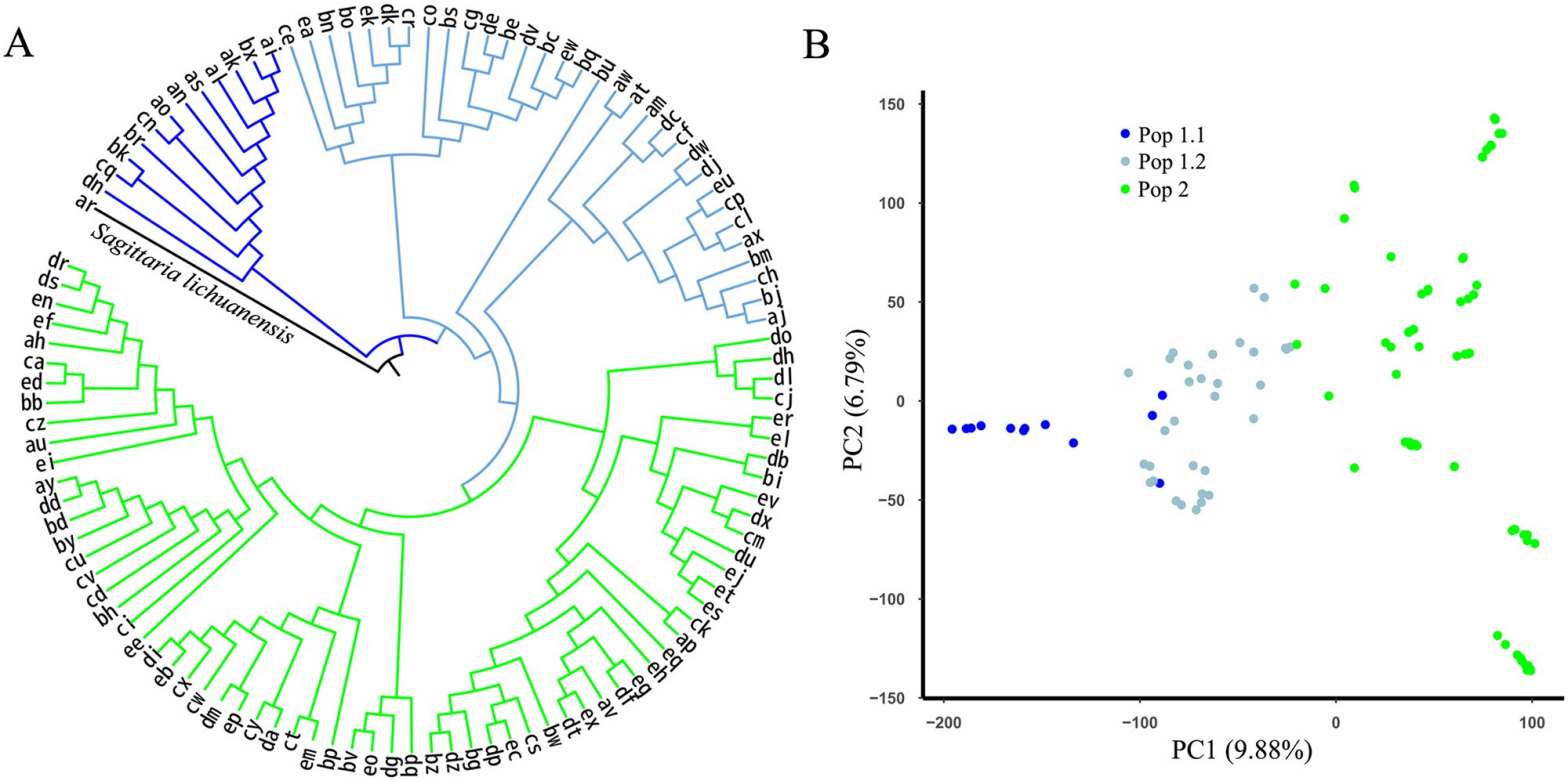
Population structure of 111 *Sagittaria trifolia* accessions. (A) Phylogenetic analysis of 111 *S*. *trifolia* accessions. Group I-1 accessions are in blue, Group I-2 accessions are in light blue, and Group II accessions are in green. (B) PCA plots of the first two components of the 111 *S*. *trifolia* accessions. The subpopulations defined by the PCA include Pop 1.1 (Group I-1), Pop 1.2 (Group I-2), and Pop 2 (Group II).

To clarify the phylogenetic relationships among all accessions, we used the NJ method in MEGA to construct a phylogenetic tree. All the accessions were classified into two groups based on their distinct geographical distribution patterns (Fig 5A, Table 2). Group I included 44 accessions, mainly from mid-southern and southwestern China, and Group II comprised 67 accessions, mainly from eastern, mid-southern, and southwestern China. Jiangsu, Zhengjiang, and Guangxi Provinces were the main arrowhead-producing areas, from which 30% of all accessions were collected. Phylogenetic relationships based on genetic distances were consistent with the assignments determined using ADMIXTURE. The PCA also separated all accessions into two major groups (Fig 5B).

**Table 2:** Geographic origin of each accession in the two groups.

Group	Total	EC	MSC	SWC	NWC	NC	The North Korea	Thailand
**I**	44	7	18	15	—	2	1	1
**II**	67	27	24	13	1	2	—	—

EC, eastern China; MSC, mid-southern China; SWC, southwestern China; NWC, northwestern China; NC, northern China.

Using *S*. *lichuanensis* as an outgroup, we explored the phylogenetic relationships among the 111 accessions. Group I was classified into two subclades: Group I-1 comprising 12 accessions and Group I-2 comprising 32 accessions. The evolutionary dynamics among acceessions of groups I-1, I-2, and II were resolved in the rooted tree (Fig 5A). Compared with the other two groups, Group I-1 was more closely related to the outgroup, revealed by the rooted tree. The evolutionary relationships among the three groups supported the direction from Group I-1 to Group I-2 and eventually to Group II. Molecular phylogenetic Group I included accessions mainly from phenotypic Cluster 1, with Group I-1 and Group I-2 exhibiting biases toward Cluster 1.1 and Cluster 1.2, respectively. Molecular phylogenetic Group II corresponded to phenotypic Cluster 2. Based on the aforementioned phenotypic analyses, there were substantial morphological differences between Cluster 1.1 and the other two clusters during the entire growth period. Although there were differences in tuber weights and leaf shapes between Cluster 1.2 and Cluster 2, these two groups were phenotypically more similar. Therefore, this information further supports the idea that accessions in Cluster 1.1 emerged first in the evolutionary process.

Similar to the phenotype-based phylogenetic tree, Group I comprised 15 landraces intermingled with 29 wild accessions, while Group II comprised 56 landraces clustered with 11 wild accessions (Fig 5A). *S*. *trifolia* with big or delicate tubers, is suitable for cultivation. Therefore, wild plants with these traits may have been collected and cultivated locally. Over time, they may have become part of the landraces. These data would suggest that the domestication period was short and the degree of domestication was low for *S*. *trifolia*; however, further research is needed to better understand the evolutionary time scale.

All landraces in this study were *S*. *trifolia* subsp. *leucopetala* and all wild resources were classified as *S*. *trifolia* subsp. *trifolia*. Twenty-nine *S*. *trifolia* subsp. *trifolia* accessions intermingled with 15 *S*. *trifolia* subsp. *leucopetala* in Group I, and another 56 *S*. *trifolia* subsp. *leucopetala* were clustered in Group II along with 11 *S*. *trifolia* subsp. *trifolia* accessions. *S*. *trifolia* resources collected from the field were classified as wild resources or landraces based on their distribution areas. After years of investigation on phenotypic traits, the collected *S*. *trifolia* accessions were classified as *S*. *trifolia* subsp. *trifolia* or *S*. *trifolia* subsp. *leucopetala* according to the “Flora of China.” Although there were differences in tuber weight, leaf shape and size, and inflorescence between the two subspecies, there were still accessions of intermediate type, making it difficult to accurately classify these accessions based on phenotypic traits. In addition, *S*. *trifolia* is a typical cross-pollinated crop [49, 50], cross-pollination can occur between these two subspecies, which can grow into new plants and produce tubers that later reproduce into large populations through asexual reproduction. Classifying of hybrid accessions based on phenotypic traits, there may be controversial. Therefore, the two subspecies examined in the present study did not cluster into two independent clades. The genetic composition of approximately 20 accessions suggests the presence of a combination of two different genetic backgrounds accounting for 50–70% and 30–50% of the total (Fig 4). Hence, gene flow must have occurred between *S*. *trifolia* subsp. *trifolia* and *S*. *trifolia* subsp. *leucopetala* and influenced the genetic integrity of *S*. *trifolia* subsp. This finding highlights the fact that accessions of intermediate types are difficult to classify based exclusively on the morphological characteristics of the accessions or SNP-based classifications.

A previous study was based exclusively on the morphological characteristics of the accessions and used a relatively small germplasm sample [46]. To date, the population structure of the arrowhead germplasm has not been investigated in depth. In the present study, we first investigated the phenotypic traits of the arrowheads and then classified the germplasm resources. There was a 14% discrepancy between the morphological classification and SNP-based phylogenetic tree. Groups I and II corresponded to clusters 1 and 2, respectively. Group I (44 accessions) included 34 accessions in Cluster 1 (39 accessions) and 10 accessions in Cluster 2 (72 accessions). Group II (67 accessions) comprised 62 accessions in Cluster 2 (72 accessions) and five accessions in Cluster 1 (39 accessions). Overall, SNP-based classification was more accurate than morphological classification because phenotypic traits were affected by environmental conditions and subjective judgments. Joint morphological and SNP-based classification analyses suggested that other phenotypic traits of the arrowhead must be investigated to identify the most important traits that differentiate the groups. Traits such as flowering time, inflorescence number, flower number in the inflorescence, seed-setting rate, germination rate, and rhizome number per plant should be considered in future research.

### Genetic diversity and population differentiation

Several genetic parameters were estimated separately to evaluate the genetic diversity and extent of differentiation among *S*. *trifolia* accessions. The global genetic diversity indices (π) for the Group I and GroupII populations were 0.26 and 0.29, respectively (Table 3). Since Group I could be further classified into different groups, we investigated the detailed relationship between groups I-1 and I-2. The diversity of Group I-1 (π = 0.21) was slightly lower than that of Group I-2 (π = 0.26). This finding was confirmed by Nei’s genetic diversity indices (0.29 for Group I-1 and 0.34 for Group I-2). The Fst values obtained from the comparison between groups I-1 and I-2 and between groups I-1 and II were 0.023 and 0.042, respectively (Table 4). A relatively low Fst (0.008) was obtained by comparing groups I-2 and II. The pairwise Nei’s genetic distance analysis between groups I-1 and II indicated the highest differentiation (0.028). Nei’s genetic distance was lowest between groups I-2 and II (0.009). Group II had wider genetic diversity than Group I-2, whereas Group I-2 had wider genetic diversity than Group I-1, which may be, in part, influenced by group size (12 Group I-1 accessions, 32 Group I-2 accessions, and 67 Group II accessions). Groups I-1 and II showed the highest differentiation, whereas groups I-2 and II were the most closely related.

**Table 3:** Summary of statistical analysis for Group I and GroupII populations based on SNP calculation.

Parameter	Group I			GroupII
	Group I-1	Group I-2	Total	
**Nucleotide diversity (π)**	0.21	0.26	0.26	0.29
**Nei’s genetic diversity index**	0.29	0.34	0.33	0.35

Groups I-1, I-2, and II are defined using the phylogenetic trees.

**Table 4:** Matrix of pairwise Nei’s genetic distance and Fst among the inferred groups.

**Inferred group**	**Group I-1**	**Group I-2**	**GroupII**
**Group I-1**	0	0.023	0.028
**Group I-2**	0.023	0	0.009
**GroupII**	0.042	0.008	0

Above diagonal: Nei’s genetic distance; below diagonal: Fst.

The overall sequence diversity levels (π) for groups I and II were 0.26 and 0.29, respectively. The global genetic diversity (π) for water dropwort *Oenanthe linearis* and *Oenanthe javanica* were 0.1902 and 0.2174, respectively. *O*. *linearis* could be further classified into linear and deep-cleft leaf types, whereas *O*. *javanica* was readily segregated into groups I and II. The value of π varied only slightly among the four water dropwort groups (range 0.1818–0.2318). Within each of the four water dropwort groups, the landraces were intermingled with the wild accessions into a single group [47]. Similar results were obtained in this study. Arrowhead is widely distributed in wild habitats and has been used as food for >1,000 years, it has been cultivated over the last 800 years, but the development of arrowhead cultivation was slow. Thus, arrowheads can now be harvested from both wild and cultivated sources as a vegetable crop.

Although Fst analysis between groups I-1 and II indicated the highest differentiation (0.042), the level of genetic differentiation between these two groups was low, which may be in part influenced by the outcrossing of *S*. *trifolia*. Both groups I-1 and I-2 accessions were inferred to have the same genetic background, and Group II accessions had different genetic backgrounds. However, the genetic differentiation between groups I-1 and I-2 was more pronounced than that between groups I-2 and II. This finding is consistent with the evolutionary relationships, as Group II accessions evolved from Group I-2 accessions. Similarly, wild rice *Oryza rufipogon* accessions were classified as Or-I, Or-II, and Or-III based on population structure analysis. The phylogenetic tree and PCA plots indicate that the cultivated rice *Oryza sativa indica* and *Oryza japonica* have descended from Or-I and Or-III, respectively. The genetic differentiation between Or-I and Or-III (Fst = 0.18) was slightly higher than that between *O*. *sativa indica* and Or-I (Fst = 0.17) [51].

## Conclusions

Phenotypic traits and SNP-based analyses were combined to investigate the phylogenetic relationships and genetic diversity of arrowheads. The SNPs generated by SLAF-seq in the present study were the most abundant markers obtained for the arrowhead. *Sagittaria trifolia* is most commonly distributed over a wide geographical area in China and its neighboring countries. SNP-based molecular characterization of the arrowhead collection examined here revealed that all accessions were categorized into three subgroups that did not fully correspond to the three subclusters determined by the phenotypic trait clustering analysis. Instead of cluster analysis of the 11 phenotypic traits, it was found that all accessions could be well clustered into three sub-clusters based mainly on the characteristics of their leaves and tubers. Using *S*. *lichuanensis* as an outgroup, the rooted phylogenetic tree revealed an evolutionary direction from Group I-1 to Group I-2 and finallly to Group II. Group II displayed higher levels of genetic diversity (π = 0.29) than group I (π = 0.26). The lowest level of population differentiation was observed between groups I-2 and II (Fst = 0.0008), supporting the hypothesis that Group II accessions evolved from Group I-2 accessions. These findings will prove highly valuable for genetic characterization, germplasm resource classification, phylogenetic relationships, and genetic diversity of arrowheads.

## Supporting information

S1 Table The germplasm set of 111 *Sagittaria trifolia* accessions and 1 *Sagittaria lichuanensis* accession: Origin, types and species. (XLSX)

S2 Table Phenotype traits of the 111 accessions of the arrowhead germplasm set. (XLSX)

S3 Table Summary of the statistical analysis of the specific-length amplified fragment (SLAF) sequencing data. (XLSX)

S4 Table Summary of the statistical analysis of the single nucleotide polymorphisms (SNPs). (XLSX)
